# Pretreatment Carcinoembryonic Antigen Level Serves as a Potential Biomarker to Guide Adjuvant Radiotherapy in pT4N+ Colon Cancer Patients

**DOI:** 10.1155/2023/4815996

**Published:** 2023-02-16

**Authors:** Dakui Luo, Ruoxin Zhang, Yufei Yang, Qingguo Li, Xinxiang Li

**Affiliations:** ^1^Department of Colorectal Surgery, Fudan University Shanghai Cancer Center, Shanghai 200032, China; ^2^Department of Oncology, Shanghai Medical College, Fudan University, Shanghai 200032, China

## Abstract

The survival benefit of adjuvant radiotherapy in T4 colon cancer (CC) remains controversial, with conflicting results reported in the literature. This study aimed to explore the relationship between pretreatment carcinoembryonic antigen (CEA) level and overall survival (OS) of pT4N+ CC patients treated with adjuvant radiotherapy. Data of pT4N+ CC patients who received curative surgery between 2004 and 2015 were identified from the Surveillance, Epidemiology, and End Results (SEER) database. The primary outcome was OS, and subgroup analysis was conducted according to pretreatment CEA level. A total of 8763 patients were eligible for our study. In the CEA-normal group, 151 patients received adjuvant radiotherapy, while 3932 patients did not. In the CEA-elevated group, 212 patients received adjuvant radiotherapy, while 4468 patients did not. In general, adjuvant radiotherapy was associated with better OS in pT4N+ CC patients (HR = 0.846, 95% CI = 0.733–0.976, *P* = 0.022). Intriguingly, only patients with an elevated pretreatment CEA level gained a survival benefit from adjuvant radiotherapy (HR = 0.782; 95% CI = 0.651–0.939; *P* = 0.008) while those with a normal pretreatment CEA level did not (HR = 0.907; 95% CI = 0.721–1.141; *P* = 0.403). Multivariable Cox regression analysis demonstrated that adjuvant radiotherapy was an independent protective factor in pT4N+ CC patients with an elevated pretreatment CEA level. Pretreatment CEA levels could serve as a potential biomarker to screen pT4N+ CC patients who would benefit from adjuvant radiotherapy.

## 1. Introduction

Colorectal cancer (CRC) is the third most prevalent malignant tumor and the second most common cause of cancer-related death worldwide [1]. Surgery-based comprehensive therapy is the gold standard for the treatment of stage III disease. Despite the fact that radiotherapy has been widely adopted for locally advanced rectal cancer, the role of radiotherapy in the management of colon cancer (CC) is largely undefined. About 10% to 15% of CC patients are diagnosed with T4 disease that penetrates the colonic serosa. The oncological outcomes remain poor for T4 CC although these patients receive multivisceral resection and adjuvant chemotherapy or radiotherapy [2]. Several retrospective studies evaluated the value of adjuvant radiotherapy in T4 CC patients, with conflicting results [3–7]. A recent study indicated that adjuvant radiotherapy did not improve survival in T4 CC using the National Cancer Database and the Surveillance, Epidemiology, and End Results (SEER) [8]. Definitely, it is unreasonable to adopt adjuvant radiotherapy for all T4 CC patients, especially those without lymph node involvement. Therefore, it is necessary to identify a subgroup that benefits more from adjuvant radiotherapy.

Carcinoembryonic antigen (CEA) is the most widely applied marker in the follow-up of CRC, and an elevated CEA level often indicates tumor relapse and poor prognosis [9–11]. Furthermore, CEA level has been found to be a potential biomarker guiding neoadjuvant or adjuvant therapy [12, 13]. Our previous study also evaluated the guiding role of an elevated pretreatment serum CEA level for adjuvant chemotherapy in stage IIA CC [14]. In our present study, we would like to determine whether pretreatment CEA level could serve as a potential biomarker to guide adjuvant radiotherapy in pT4N+ CC patients.

## 2. Methods

### 2.1. Patient Selection

This is a retrospective cohort study. pT4N + M0 CC (AJCC 7th edition) patients who received curative surgery and had the definite data regarding pretreatment CEA status and adjuvant radiotherapy were recruited from the SEER database (2004–2015). The SEER database (https://seer.cancer.gov/seerstat/) was an authoritative and public source of information on cancer incidence, mortality, prevalence, lifetime risk statistics, and survival among 26% of the population in 18 cancer registries in the United States. In general, the inclusion criteria were detailed as follows: patients were diagnosed with pT4N + M0 CC; patients did not receive neoadjuvant radiotherapy; curative surgery was performed; patients had a definite CEA status; survival data were available. The exclusion criteria were detailed as follows: patients with distant metastases; patients who received both neoadjuvant radiotherapy and adjuvant radiotherapy. The following pathological and survival data were collected: histologic type, grade, tumor size, node stage, radiotherapy, pretreatment serum CEA level, and survival data. Overall survival (OS) was regarded as a primary endpoint. OS was defined as the time from the date of diagnosis to the date of death.

### 2.2. Statistical Analysis

The differences of categorical variables between two groups were determined by *χ*^2^ test. The Kaplan–Meier (K−M) method and the univariate Cox regression model were utilized for comparison of the survival difference between the two groups with a log-rank test. A multivariate Cox regression model was used to seek for the independent prognostic factors. All statistical analyses were performed using SPSS 25.0.

## 3. Results

### 3.1. Patients' Characteristics

A total of 8763 patients were eligible for our study. 363 patients received adjuvant radiotherapy, and 8400 patients did not receive adjuvant radiotherapy. In CEA-normal group, 151 patients received adjuvant radiotherapy, while 3932 patients did not. In the CEA-elevated group, 212 patients received adjuvant radiotherapy, while 4468 patients did not. The baseline characteristics between two groups stratified by CEA status are summarized in Table 1.

### 3.2. Survival Benefit of pT4N + M0 CC Patients from Adjuvant Radiotherapy

**Figure 1:**
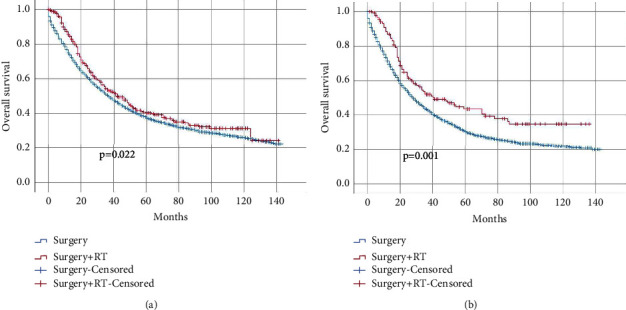
Kaplan–Meier curves for overall survival for CC patients who received surgery and surgery plus adjuvant radiotherapy. (a) pT4N + M0 patients; (b) pT4N2M0 patients.

First, we evaluated the role of adjuvant radiotherapy in pT4N + M0 CC patients. As expected, adjuvant radiotherapy was associated with better oncological outcomes (HR = 0.846; 95% CI: 0.733–0.976; *P*=0.022) (Figure 1(a)). Remarkably, pT4N2M0 CC patients gained more survival benefit from adjuvant radiotherapy (HR = 0.683; 95% CI: 0.542–0.861; *P*=0.001). Kaplan−Meier curves are plotted (Figure 1(b)).

### 3.3. Association of Pretreatment CEA Status and Adjuvant Radiotherapy in Predicting Prognosis

**Figure 2:**
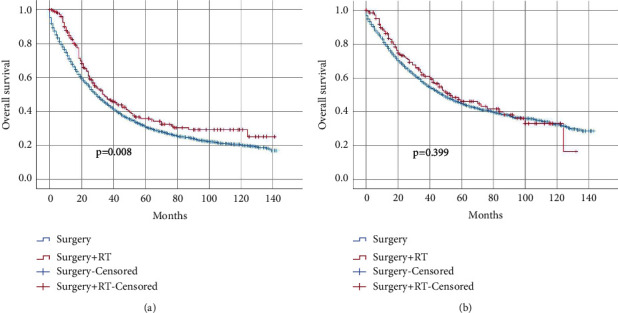
Kaplan–Meier curves for overall survival for pT4N + M0 CC patients who received surgery and surgery plus adjuvant radiotherapy stratified by pretreatment CEA level. (a) Elevated CEA level; (b) normal CEA level.

Next, we wonder whether serum CEA status had a guiding role in predicting a survival benefit from adjuvant radiotherapy. We found that receipt of adjuvant radiotherapy was associated with better oncological outcomes in the CEA-elevated group (5-year OS: 31.0% VS 35.9%, log-rank test, *P* = 0.008; HR = 0.782, 95% CI = 0.651–0.939, *P* = 0.008) (Figure 2(a)). Similarly, after adjusting for other pathological prognostic factors, the results of multivariate Cox analysis indicated that adjuvant radiotherapy significantly decreased mortality risk in the CEA-elevated group (HR = 0.781; 95% CI: 0.649–0.938; *P* = 0.008, Table 2). On the contrary, receiving or not receiving adjuvant radiotherapy did not presented a survival difference statistically in CEA-normal group (5-year OS: 44.8% vs. 46.0%, log-rank test, *P* = 0.399; HR = 0.907, 95% CI = 0.721–1.141, *P* = 0.403) (Figure 2(b)).

## 4. Discussion

In the current study, we demonstrated that pT4N + M0 CC patients could benefit from adjuvant radiotherapy. Especially, only pT4N + M0 CC patients with elevated pretreatment CEA level obtained a survival benefit from adjuvant radiotherapy while those with normal pretreatment CEA level did not. Further multivariable Cox regression analysis demonstrated that adjuvant radiotherapy was an independent protective factor in pT4N+ CC patients with an elevated pretreatment CEA level.

There has been controversy over adopting adjuvant radiotherapy for locally advanced CC. Despite this, current NCCN guidelines recommend receipt of radiotherapy for T4 CC patients. In earlier research, postoperative radiotherapy appeared to improve local control in T4 CC patients [3, 4]. In the modern chemotherapy era, evidence indicated that the benefits of locoregional control and enhanced disease-free survival were still observed for T4 colon cancer treatment with adjuvant radiotherapy [5]. A prospective, randomized controlled trial (Intergroup-0130) evaluated the role of radiotherapy in resected CC but failed to meet its accrual goals [6]. Recently, Margalit et al. reported that postoperative radiation did not improve overall survival in individuals with pathologic stage T4 colon cancer using the National Cancer Database [7]. Specifically, T4b colon tumors also did not obtain a survival benefit from adjuvant radiotherapy, regardless of surgical margin status. Similarly, another large comparative study also demonstrated that T4 CC patients did not benefit from adjuvant radiotherapy using the National Cancer Database and the Surveillance, Epidemiology, and End Results Program [8]. It is interesting to note whether a specific subgroup is likely to benefit from adjuvant radiotherapy. Adjuvant radiotherapy is currently recommended for node-positive middle and low-grade rectal cancer, which does not receive neoadjuvant radiotherapy to reduce the local recurrence rate. We wondered whether adjuvant radiotherapy improved survival in pT4 node-positive CC patients. Our study demonstrated that adjuvant radiotherapy resulted in a decreased risk of mortality. Consistently, Huang et al. found that adjuvant radiotherapy could decrease CC-related mortality risk by nearly 30% in the T4N2M0 subgroup, which was significantly higher than the 11.51% of the general T4 population [15].

Pretreatment CEA level has been identified as a predictive biomarker of treatment response. Wang et al. found that the pretreatment CEA level may be considered a potential biomarker to select locally advanced rectal cancer patients who would benefit from preoperative radiotherapy [16]. Huang et al. reported that pretreatment serum CEA level may serve as a promising biomarker guiding adjuvant chemotherapy in rectal cancer patients with ypT3N0M0 following neoadjuvant radiotherapy [17]. These studies indicated that the pretreatment CEA level could be used as a determinant for adopting neoadjuvant radiotherapy. Although adjuvant radiotherapy for pT4 node-positive patients offers a survival advantage, it is unreasonable to adopt adjuvant radiotherapy for all pT4 node-positive CC patients in terms of therapeutic toxicity and treatment cost. It is valuable to identify a subgroup that benefits more from adjuvant radiotherapy. Intriguingly, our results indicated that adjuvant radiotherapy would only benefit pT4N+ CC patients with an elevated pretreatment CEA level. This finding suggested that pretreatment CEA level could be used as a potential biomarker to select pT4N+ CC patients who would gain the long-term survival benefit from adjuvant radiotherapy. To the best of our knowledge, this is the first time in the literature that we have found a biomarker to guide adjuvant radiotherapy in T4N+ CC patients. It is particularly important for pT4N+ CC patients with a normal pretreatment CEA level to avoid the side effects of radiotherapy without compromising oncological outcomes.

However, there are several inevitable limitations to the present study. First, selection bias is hard to avoid in a retrospective analysis. Second, data regarding serum CEA level after surgery were unavailable in the SEER database. Besides that, data regarding the circumferential resection margin and tumor deposits in the SEER database were unavailable between 2005 and 2009. Thus, we did not include these important parameters for further analysis. Third, it is impossible to recognize T4a or T4b from this subgroup.

## 5. Conclusions

In conclusion, pretreatment CEA level could serve as a potential biomarker to identify pT4N+ CC patients who benefited from adjuvant radiotherapy.

## Figures and Tables

**Table 1:** Comparison of clinicopathological features according to receipt of adjuvant radiotherapy, stratified by the CEA status.

Variable	Elevated CEA level			Normal CEA level		
	Surgery + RT	Surgery	*P*	Surgery + RT	Surgery	*P*
Age (years)			<0.001			<0.001
≤60	104	1251		71	1270	
>60	108	3217		80	2662	
Sex			0.006			0.926
Male	115	1994		77	1990	
Female	97	2474		74	1942	
Histologic type			0.117			0.703
Adenocarcinoma	156	3259		110	2950	
Mucinous	38	623		18	491	
Signet ring cell	4	170		5	134	
Other	14	416		18	357	
Grade			0.047			0.338
G1 + G2	134	2564		88	2208	
G3 + G4	69	1788		58	1651	
Unknown	9	116		5	73	
Tumor size			<0.001			0.003
<5 cm	57	1961		56	2018	
≥5 cm	143	2326		89	1796	
Unknown	12	181		6	118	
N stage			0.007			0.394
N1	135	2422		91	2232	
N2	77	2046		60	1700	

**Table 2:** Univariate and multivariate Cox analyses of the clinicopathological factors for overall survival in pT4N + colon cancer patients with elevated pretreatment CEA levels.

Variable	Univariate analysis		Multivariate analysis	
	HR (95% CI)	*P* value	HR (95% CI)	*P* value
Histologic type
Adenocarcinoma	Reference		Reference	
Mucinous	1.081 (0.973–1.202)	0.148	1.048 (0.940–1.167)	0.398
Signet ring cell	1.559 (1.304–1.864)	<0.001	1.306 (1.086–1.571)	0.005
Other	—		—	
Grade
G1 + G2	Reference		Reference	
G3 + G4	1.307 (1.211–1.410)	<0.001	1.234 (1.140–1.335)	<0.001
Unknown	—		—	
Tumor size
<5 cm	Reference		Reference	
≥5 cm	1.113 (1.030–1.202)	0.006	1.087 (1.005–1.175)	0.036
Unknown	—		—	
N stage
N1	Reference		Reference	
N2	1.225 (1.137–1.319)	<0.001	1.195 (1.109–1.289)	<0.001
Radiotherapy
Negative	Reference		Reference	
Positive	0.782 (0.651–0.939)	0.008	0.781 (0.649–0.938)	0.008

## Data Availability

The data that support the findings of this study are available from the corresponding author upon reasonable request.
